# Investigation of genetic relationships within three *Miscanthus* species using SNP markers identified with SLAF-seq

**DOI:** 10.1186/s12864-021-08277-8

**Published:** 2022-01-10

**Authors:** Zhiyong Chen, Yancen He, Yasir Iqbal, Yanlan Shi, Hongmei Huang, Zili Yi

**Affiliations:** 1 College of Bioscience & Biotechnology, Hunan Agricultural University, Changsha, 410128 PR China; 2 Hunan Engineering Laboratory of Miscanthus Ecological Applications, Hunan Agricultural University, Changsha, 410128 PR China

**Keywords:** *Miscanthus*, SLAF-seq, SNP, SLAF tags, High-throughput sequencing, Identification of genetic relationship

## Abstract

**Background:**

*Miscanthus,* which is a leading dedicated-energy grass in Europe and in parts of Asia, is expected to play a key role in the development of the future bioeconomy. However, due to its complex genetic background, it is difficult to investigate phylogenetic relationships in this genus. Here, we investigated 50 *Miscanthus* germplasms: 1 female parent (*M. lutarioriparius*), 30 candidate male parents (*M. lutarioriparius, M. sinensis,* and *M. sacchariflorus*), and 19 offspring. We used high-throughput Specific-Locus Amplified Fragment sequencing (SLAF-seq) to identify informative single nucleotide polymorphisms (SNPs) in all germplasms.

**Results:**

We identified 257,889 SLAF tags, of which 87,162 were polymorphic. Each tag was 264–364 bp long. The obtained 724,773 population SNPs were used to investigate genetic relationships within three species of *Miscanthus.* We constructed a phylogenetic tree of the 50 germplasms using the obtained SNPs and grouped them into two clades: one clade comprised of *M. sinensis* alone and the other one included the offspring, *M. lutarioriparius,* and *M. sacchariflorus*. Genetic cluster analysis had revealed that *M. lutarioriparius* germplasm C3 was the most likely male parent of the offspring.

**Conclusions:**

As a high-throughput sequencing method, SLAF-seq can be used to identify informative SNPs in *Miscanthus* germplasms and to rapidly characterize genetic relationships within this genus. Our results will support the development of breeding programs with the focus on utilizing *Miscanthus* cultivars with elite biomass- or fiber-production potential for the developing bioeconomy.

## Background

*Miscanthus,* a high-biomass-yielding perennial C4 grass, has great potential utility for biobased industry [1–4]. This is a heterogeneous gramineous plant that hybridizes interspecifically and hence has a complex genetic background. Recent molecular studies have revealed that the *Miscanthus* genome is unusually large (2.07–2.6 GB) [5–9]. In *Miscanthus*, inter- and intraspecific hybridization is common which is why this genus is characterized by rich genetic diversity and heterosis [6, 9]. The genetic diversity has been exploited to develop *Miscanthus* hybrids, which can deliver higher biomass yields and exhibit better adaptability to diverse climatic conditions than their parent species. The improved biomass yields of hybrids are attributed to efficient rates of photosynthesis [3]. On the other hand, this high degree of genetic diversity also increases the complexity of interspecific relationships in the *Miscanthus* and subsequently makes it challenging to carry out the genetic evolution analyses of this genus. Thus, it is difficult to mine functional genes in *Miscanthus* which can seriously limit the utilization potential of *Miscanthus* [5]. Molecular markers would be useful for further investigations of *Miscanthus* plants because such markers have been widely used in studies of genetics, molecular population genetics, species formation, evolutionary and phylogenetic relationships [10–12], and molecular taxonomy [6].

First-generation molecular markers cover restriction fragment length polymorphisms (RFLPs) [7, 8], random amplified polymorphic DNA (RAPD) [9, 10], and amplified fragment length polymorphisms (AFLPs) [11], while second-generation molecular markers include simple sequence repeats (SSRs) [12] and inter-simple sequence repeats (ISSRs) [13]. However, these markers have several limitations: they are low throughput, inaccurate, time-consuming, labor-intensive, and costly [3]. These drawbacks have motivated the development of third-generation polymorphic molecular markers, which are named as SNPs. These markers are generally widely distributed throughout the whole genome [14]. SNP markers are amenable to large-scale automated monitoring and have been instrumental in various crop breeding programs, such as the construction of genetic maps, the DNA fingerprinting of germplasm resources, the detection of molecular biodiversity, and the analysis of linkage disequilibrium [15]. This continuous development of molecular marker technology has accelerated functional gene identification and characterization in other crops, which has led to the development of varieties with improved functional traits [5]. Thus, these techniques might be useful for molecular genetic research in *Miscanthus*.

Although genotyping-by-sequencing (GBS) and restriction site-associated DNA sequencing (RAD-seq) have been used extensively in *Miscanthus*, there are still some difficulties and challenges associated with the application of these techniques in *Miscanthus* [16]. For example, one obstacle in the widespread use of GBS is the difficulty in carrying out the associated bioinformatics analysis, which is typically hampered by a large number of erroneous SNP interferences that are not easy to diagnose or correct [17].

To overcome these challenges, we aimed to develop and identify SNP markers for *Miscanthus* using SLAF-seq techniques. Moreover, reduction in the genomic complexity using specific digestion, develop markers via the high-throughput sequencing of representative libraries, and determine phylogenetic relationships using genotyping is also part of this study.

SLAF-seq uses bioinformatics methods to systematically analyze known genome sequences as well as of related species, bacterial artificial chromosome (BAC) sequences, or Fosmid sequences [18–22]. SLAF-seq techniques differ in several ways from GBS or RAD-seq techniques. The major differences cover: a) SLAF-seq identifies one tag about every 10 K; b) SLAF tags are uniformly distributed, which means that important chromosome segments are not missed; c) SLAF-seq avoids repetitive sequences, which makes it a cost effective technique. Moreover, such SLAF-seq utilizes deep sequencing to ensure genotyping accuracy, a pre-designed representation scheme to optimize marker efficiency and a double-barcode system for large populations [23].

Due to its efficient identification of SNP markers, SLAF-seq has been widely used in variety of crops [24–28] for the development of specific molecular markers and genetic maps [29]. For example, Sun et al. used 50,530 SLAFs with 13,291 SNPs to genotype the F1 population of the common carp [30]. SLAF-seq was also used to develop the first high-density genetic maps for several economically important species, including sesame [31], cucumber [32], the brown alga *Undaria pinnatifida* (Phaeophyceae) [33], wax gourd [34], watermelon (*Citrullus lanatus* L.) [35], tobacco [36], soybean [37], peanut [37], and *Salvia miltiorrhiza* [38]. In addition, an increasing number of studies for the Gramineae family have been performed using SLAF-seq [25, 27, 28, 39–41]. For example, SLAF-seq was used to develop the first 7E-chromosome-specific molecular markers for *Thinopyrum elongatum* [39], while 5142 polymorphic SLAFs were analyzed to identify a new maize inflorescence meristem mutant [40]. Zhang et al. used 69,325 high-quality SLAFs, of which 26,248 were polymorphic, to develop sufficient markers for a segregating *Agropyron* F1 population [28]. Furthermore, a high-density genetic linkage map for orchardgrass was developed using 2467 SLAF markers and 43 SSR markers [25], and in barley the semi-dwarf gene was fine-mapped using molecular markers developed with SLAF-seq [27]. The successful application of SLAF-seq in other species provides reference material for the development of SNPs in this study.

Genome-wide SLAF markers and SNPs for the three *Miscanthus* species are generated by using SLAF-seq as a part of current study. In addition, phylogenetic relationships are estimated with these species based on the generated SNPs. The genome-wide markers for *Miscanthus* identified in this study will lead towards the utilization of its genetic resources to develop molecular marker-assisted *Miscanthus* breeding programs.

## Results

### Evaluation of the digestive enzymes

Enzyme digestion was performed according to set selection principles which included: a) the proportion of restriction fragment located in the repeat sequence was low; b) the fragments were evenly distributed in the control genome; c) the length of restriction fragment was appropriate for the experimental system; d) the number of SLAF tags was consistent with the expected number of tags. The pair-end digestion efficiency of *Eco*RV + *Sca*I for the control genome (Nipponbare) was 90.87%, indicating that this enzyme combination was suitable.

### Analysis of the SLAF-seq and SNP data

We obtained 57.8 Mb clean sequence reads based on SLAF library construction and high-throughput sequencing. The average sequencing depth per sample was 11.76x for the female parent (sample A12), whereas 15.47x for the male parents (samples A1–A11, B1–B10, and C1–C9), and 7.85x for the progeny (samples D1–D19). The average GC content across all sequences was 41.39%. Across all sequences, the average number of bases with a quality score ≥ 30 (Q30) was 93.66%. In parallel, we obtained 7.17 Mb reads by sequencing the rice genome, which indicated that the experimental database was accurate.

**Fig. 1:**
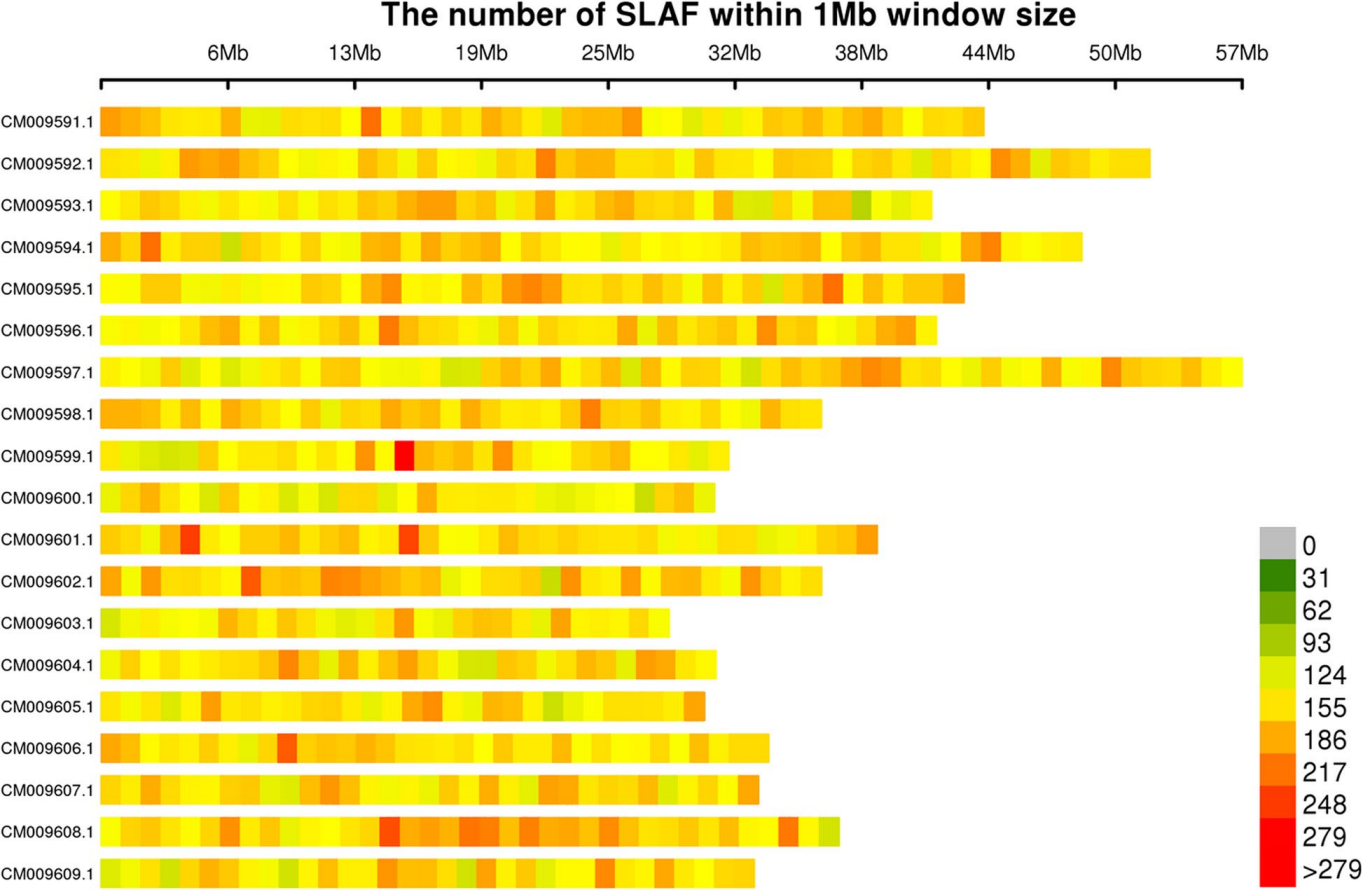
Distribution of SLAF tags on *Miscanthus* chromosomes. Chromosome length is shown on the abscissa: each band represents a chromosome, and the genome is divided into 1 Mb segments. Each segment is colored according to the number of SLAF tags: the darker areas are those where SLAF tags are concentrated

Using the obtained clean sequence reads, we developed 257,889 SLAF tags, of which 87,162 were polymorphic. We also generated a map showing the distribution of SLAF tags across the *Miscanthus* chromosomes based on the chromosome-level of reference genome data for *M. lutarioriparius* [8] (Fig. 1).

**Fig. 2:**
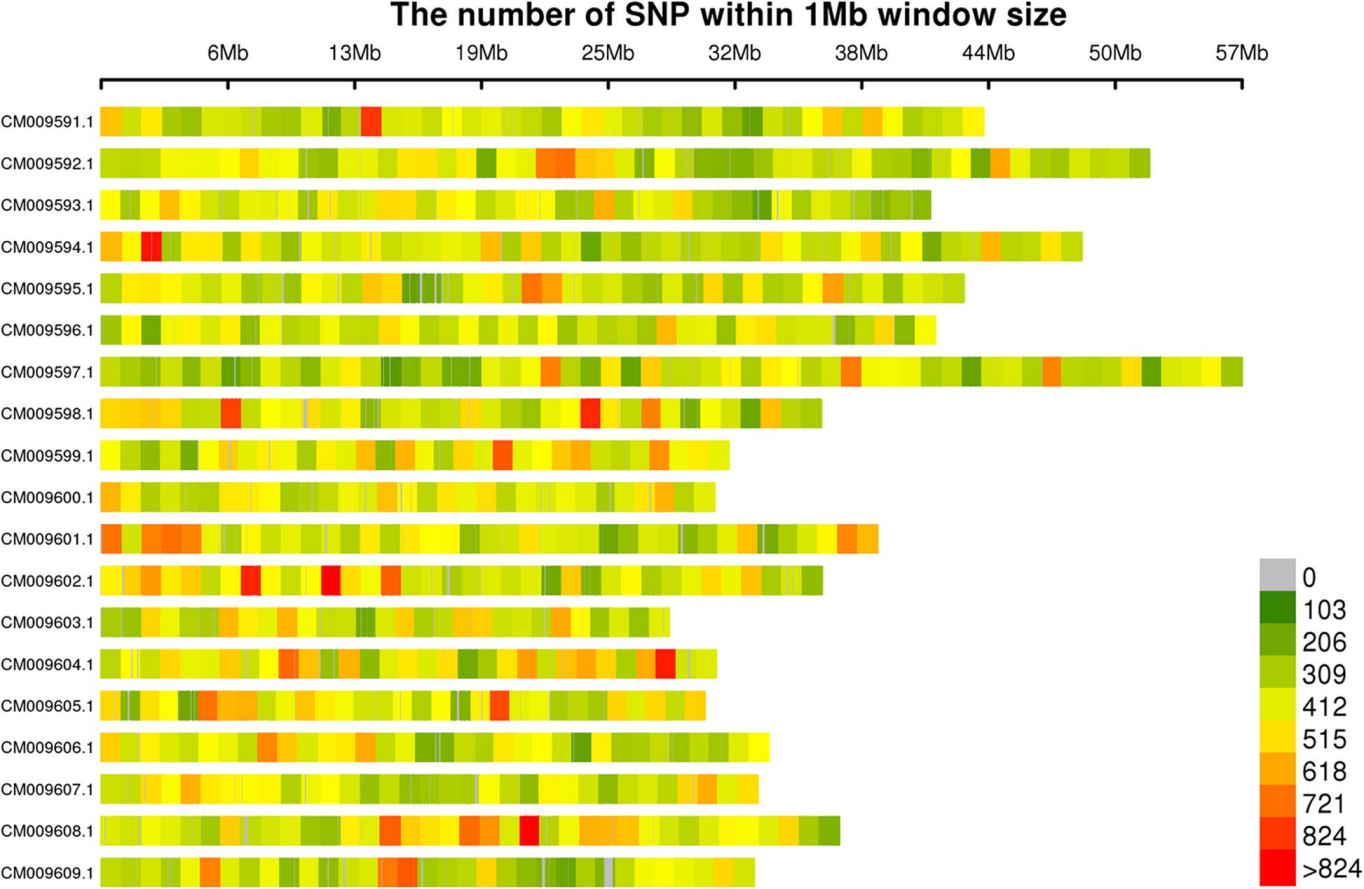
Map showing the distribution of SNPs across *Miscanthus* chromosomes. Chromosome length is shown on the abscissa: each band represents a chromosome, and the genome is divided into 1 Mb segments. Each segment is colored according to the number of SNPs: the darker areas are those where SNPs are concentrated

In total, 724,773 highly consistent population SNPs with integrity of > 0.8 and minor allele frequency (MAF) of > 0.05 were identified across all samples in this study. A map showing the distribution of these SNPs across the *Miscanthus* chromosomes is presented in Fig. 2.

### Genetic diversity of three *Miscanthus* species

**Fig. 3:**
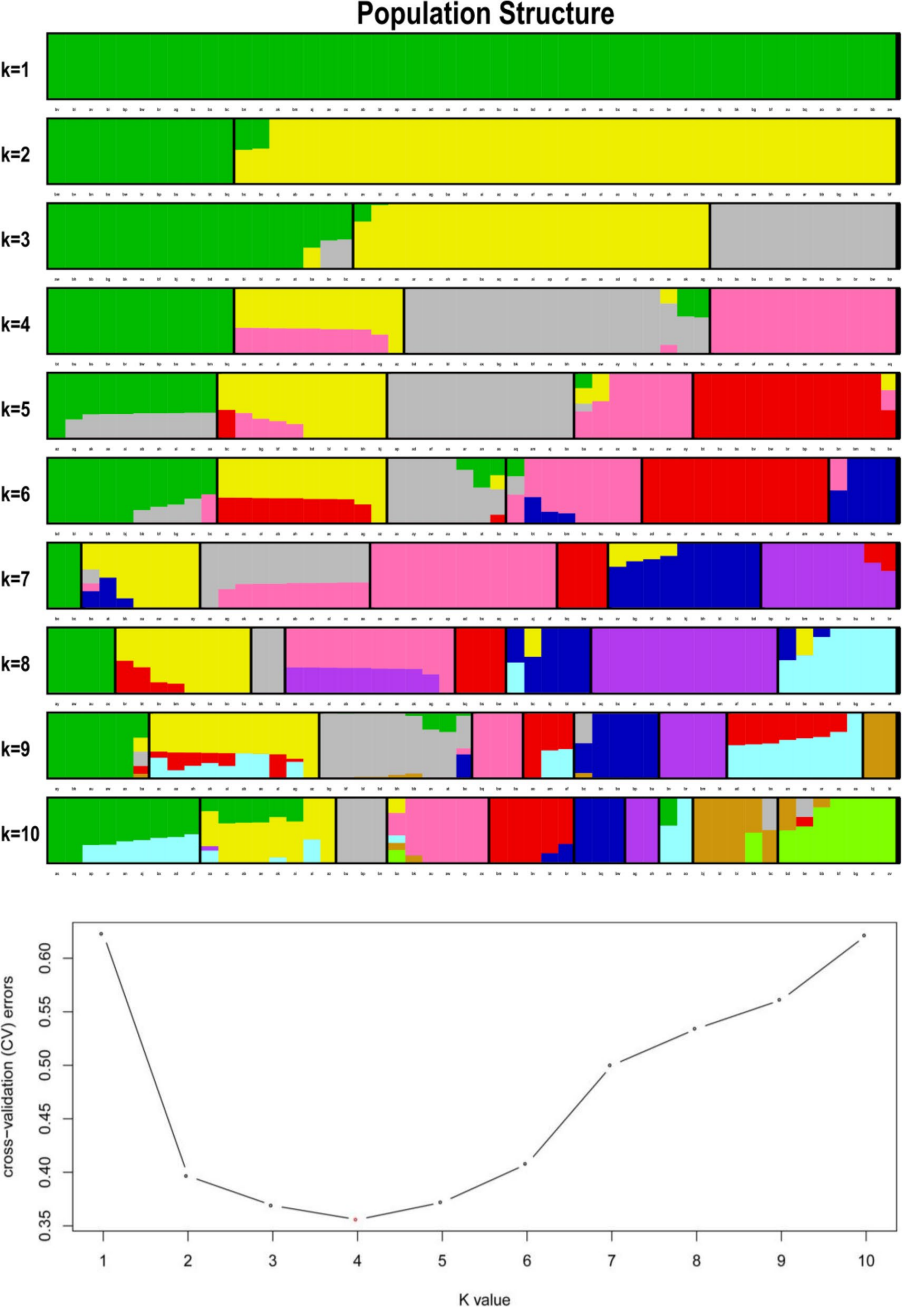
Cluster graphs of K = 1–10 with 50 samples. Each color in the figure to the left represents a cluster, with each row representing a cluster value. In the figure to the right, ΔK is the value corresponding to each K value. ΔK was minimized when K = 4

Genetic diversity analyses can provide information about the origin and composition of individual lineages. In this study, we analyzed the genetic diversity of three *Miscanthus* species based on SNPs. First, population structure was analyzed under the assumption that the number of clusters (K) were ranged 1–10. At a minimum value of ΔK, there were four clusters, suggesting that all of our samples may have originated from four primitive ancestors. Cluster graphs showing K values of 1–10 of 50 samples are shown in Fig. 3. The 50 samples clustered into four groups: the A group included A1–A11; the B group included B1–B10, C1, C2, and C4–C9; the C group included C3, D8, D9, D11–D13, D15, and D17–D19; and the D group included A12, D1–D7, D10, D14, and D16.

**Fig. 4:**
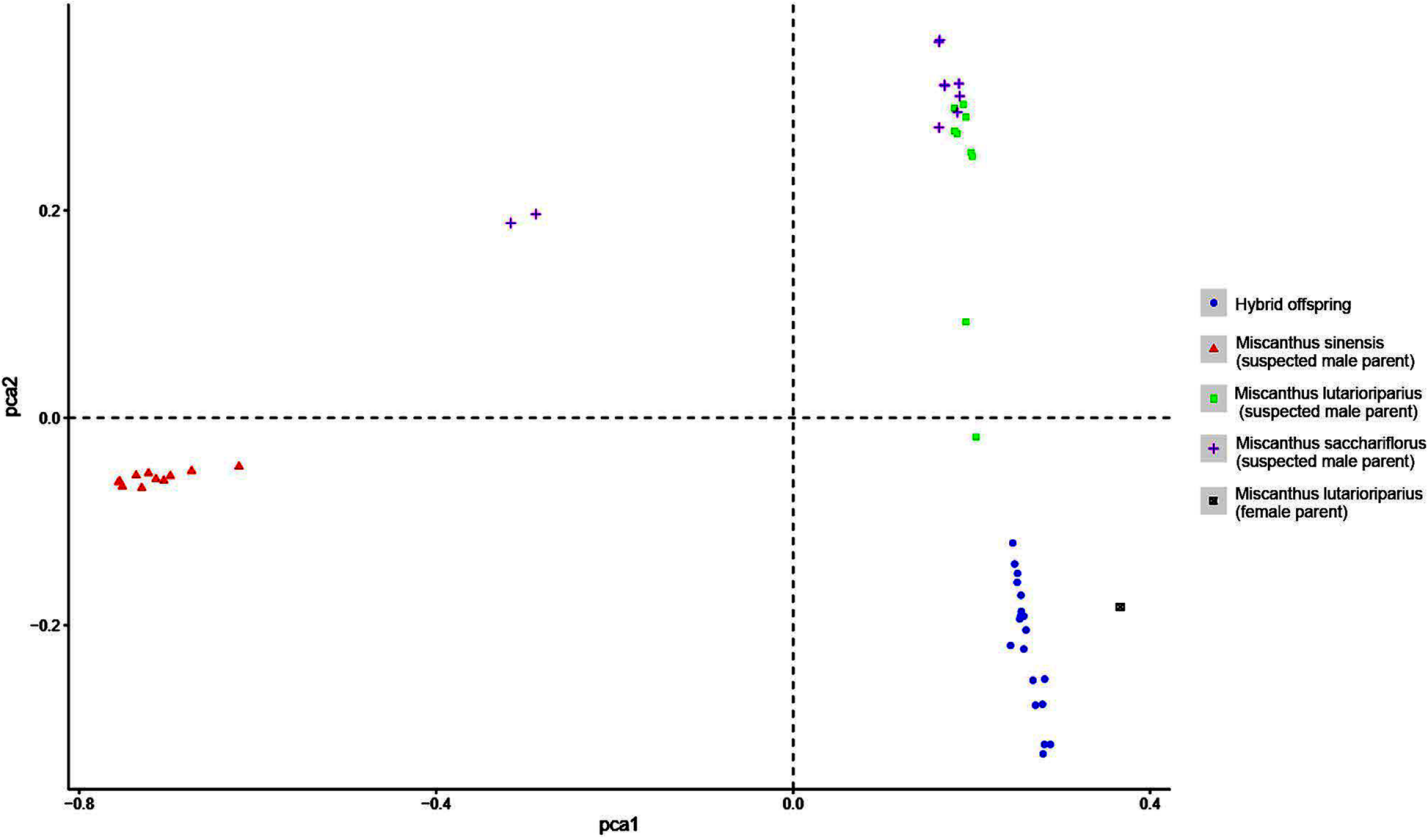
Principal component analysis (PCA) of the 50 *Miscanthus* germplasms. Each point represents a sample, and points of different color and shape correspond to different sample groups

PCA analysis indicated that the *M. sinensis* (A1-A11) germplasms were distinct from all other groups. The offspring (D1-D19), the female parent A12 (*M. lutarioriparius)*, and sample C3 (*M. lutarioriparius*) formed a loose cluster (Fig. 4). The remaining *M. lutarioriparius* and most of the *M. sacchariflorus* formed a cluster, as did B8 and B10 (*M. sacchariflorus*, Fig. 4).

**Fig. 5:**
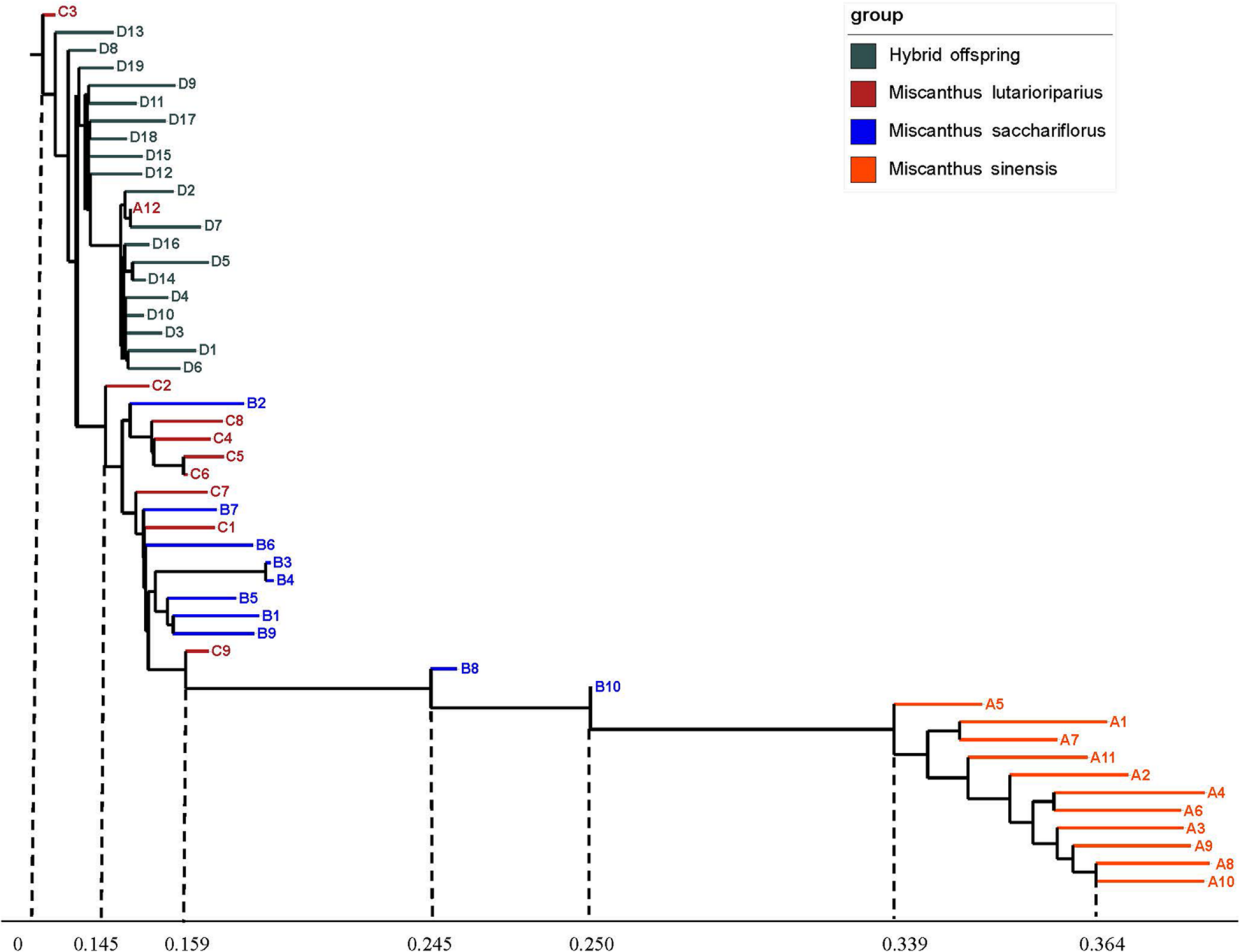
Neighbor-joining phylogenetic analysis of all 50 *Miscanthus* germplasms. The *M. lutarioriparius* genome was used as the reference. Each node represents a sample. Scale bar corresponds to genetic distance

The phylogenetic analysis indicated that the investigated 50 accessions fell into three distinct groups: 1) group containing the accessions from *M. sinensis* (A1-A11); 2) containing the accessions from A12, C3, and all offspring; 3) this group containing the accessions from *M. lutarioriparius* and *M. sacchariflorus* except B8 and B10 (Fig. 5).

The results of phylogenetic analysis showed that the genetic variation within some populations was greater than between populations. Furthermore, population structure analysis, PCA, and phylogenetic analysis indicated that C3 was the male parent of the offspring.

## Discussion

### Optimization of molecular markers based on SLAF-seq and SNPs in *Miscanthus*

Currently, supply of sufficient quantities of sustainably produced biomass with optimal quality characteristics is a major challenge in the development of biobased industry. Thus, genetic improvements to deliver high biomass yield with required quality traits can be a way forward. Biomass quality may be substantially improved by the development of genetic markers associated with quality traits [31]. SNPs, which are more abundant in the genome than any other molecular markers, are particularly useful for the analyses of genetic diversity and population structure [33]. In this study, we used SLAF-seq to efficiently identify SNP markers. Compared with other methods, such as GBS and RAD-seq, SLAF-seq is more accurate, faster, and less expensive. Moreover, SLAF-seq also reduces genome complexity [34]. Here, we obtained 257,889 SLAF tags and 724,773 SNPs, which is greater than the number of SNPs previously obtained in the *Miscanthus* genome using RAD-seq [35]. In addition, SLAF and SNP markers were evenly distributed on each chromosome based on the chromosome-level of the reference genome of *M. lutarioriparius* (Figs. 1, 2). These polymorphic molecular markers are highly discriminatory and can be used for genetic map construction and gene mapping in *Miscanthus*.

### Phylogenic analysis based on *Miscanthus* SNPs

The heterozygosity and polyploidy that have accumulated in *Miscanthus* genomes over their long evolutionary history make it more difficult to sequence complete genomes in this genus [5]. Fortunately, the genomes of *M. sinensis*, *M. lutarioriparius*, *M. sacchariflorus,* and *M. floridulus* have been sequenced through the joint efforts of many researchers [6–8]. With these reference genomes, the accuracy of *Miscanthus* phylogenetic analyses can be further improved through SNPs.

In this study, the *M. lutarioriparius* was selected as the reference genome. The use of the *M. lutarioriparius* reference genome enabled us not only to cluster the 50 samples, but also to draw a rooted phylogenetic tree (Figs. 5, 6). The phylogenetic tree in combination with the morphological characteristics indicated that the offspring had been produced by the intraspecific hybridization of *M. lutarioriparius*, which was also consistent with previous studies [8, 11, 15]. The phylogenetic analyses revealed that *M. lutarioriparius* is adjacent to *M. sacchariflorus* and had shown that the coefficient of intraspecific genetic variation in these two species was high.

It had been believed for long that the distribution of *M. lutarioriparius* is extremely narrow, consisting of only the middle and lower shallows of the Yangtze River in Southern China [42]. However, in recent years, latest studies had revealed that *M. lutarioriparius* is extremely adaptable with diverse geographic distribution and can even thrive in marginal areas such as saline alkali soils [43] and arid conditions [44]. In addition, the recorded distribution of *M. lutarioriparius* now includes both shallows and wetlands [45]. *M. sacchariflorus* is considered highly adaptable [7], thus this diverse geographic distribution of *M. lutarioriparius* can be explained through the genetic similarity between *M. lutarioriparius* and *M. sacchariflorus*. The latest studies had shown that *M. lutarioriparius* and *M. sinensis* diverged very recently [8]. This argument can be supported by the interspecific genetic distance between *M. lutarioriparius* and *M. sinensis*, which was even lower than that within *M. lutarioriparius*.

## Conclusions

We obtained 724,773 SNPs using SLAF-seq technology. We successfully identified the paternal parent and obtained an intraspecific hybrid polyploid population of *M. lutarioriparius*. Despite the high similarity between the genomes, *M. lutarioriparius* is morphologically distinct from *M. sacchariflorus*. The polyploid *M. lutarioriparius* stands out as an excellent strain, which produces more biomass and is highly adaptable than any other *Miscanthus* species, including the diploid *M. sacchariflorus*. Based on the SNPs obtained for this population as a part of this study, high-density molecular marker linkage maps could be constructed. Such maps would be valuable genetic resource for the development of miscanthus based bioeconomy. Our results will also support future genetic improvements in biomass yield as well as quality traits in *Miscanthus* species.

## Methods

### Plant materials

A total of 50 *Miscanthus* specimens were used in this study: the maternal plant *Miscanthus lutarioriparius* (A12; tetraploid, 2n = 4x), the paternal candidate *M. sinensis* (A1–A11; diploid, 2n = 2x), the paternal candidate *M. saccariflorus* (B1–B10; diploid, 2n = 2x)*,* the paternal candidate *M. lutarioriparius* (C1–C9; diploid, 2n = 2x), and 19 progenies (D1–D19; triploid, 2n = 3x). The 19 progenies were generated via open pollination from A12. A12, which was bagged during flowering to test its self-pollination seed-setting rate. The measured self-pollinating seed-setting rate was 0, indicating that A12 was highly self-incompatible. Other *Miscanthus* genotypes that flowered simultaneously with A12 in the Germplasm Nursery were selected as paternal candidates. The collection of maternal and paternal candidate materials were collected from across China (Table 1). All the plants were grown in the *Miscanthus* Germplasm Nursery at Hunan Agricultural University, Changsha, Hunan, China (latitude 28°11′, longitude 113°04′).

**Table 1:** Origins of the 31 pure species *Miscanthus* germplasms used in this study. The female parent is sample A12; the remaining samples are the candidate male parents. All collection sites are in China

Sample ID	Species	Latitude	Longitude	Altitude (m)	Collection site
A12	*M. lutarioriparius*	N32°37′	E119°14′	28	Nanjing, Jiangsu
A1	*M. sinensis*	N28°28′	E109°53′	531	Yongshun, Hunan
A2	*M. sinensis*	N29°28′	E113°17′	103	Yueyang, Hunan
A3	*M. sinensis*	N28°56′	E115°49′	30	Yongxiu, Jiangxi
A4	*M. sinensis*	N31°21′	E110°36′	500	Shennong, Hubei
A5	*M. sinensis*	N26°33′	E106°28′	1480	Qingzheng, Guizhou
A6	*M. sinensis*	N31°06′	E110°47′	303	Xinshan, Hubei
A7	*M. sinensis*	N29°35′	E103°37′	505	Leshan, Sichuan
A8	*M. sinensis*	N36°29′	E117°50′	290	Zibo, Shandong
A9	*M. sinensis*	N31°36′	E114°16′	109	Dawu, Hubei
A10	*M. sinensis*	N31°11′	E115°43′	720	Jinzhai, Anhui
A11	*M. sinensis*	N30°42′	E118°26′	88	Jingxian, Anhui
B1	*M. sacchariflorus*	N36°17′	E113°11′	908	Changzhi, Shanxi
B2	*M. sacchariflorus*	N43°23′	E126°53′	257	Huadian, Jilin
B3	*M. sacchariflorus*	N41°24′	E123°58′	136	Benxi, Liaoning
B4	*M. sacchariflorus*	N40°11′	E124°17′	50	Dandong, Liaoning
B5	*M. sacchariflorus*	N33°54′	E113°55′	82	Linying, Henan,
B6	*M. sacchariflorus*	N31°36′	E114°16′	106	Dawu, Hubei
B7	*M. sacchariflorus*	N31°47′	E117°31′	37	Hefei, Anhui
B8	*M. sacchariflorus*	N30°32′	E118°27′	113	Jingxian, Anhui
B9	*M. sacchariflorus*	N37°46′	E118°59′	0	Dongying, Shandong
B10	*M. sacchariflorus*	N36°29′	E117°50′	280	Zibo, Shandong
C1	*M. lutarioriparius*	N32°59′	E120°48′	2	Dafeng, Jiangsu
C2	*M. lutarioriparius*	N32°59′	E119°55′	3	Taizhou, Jiangsu
C3	*M. lutarioriparius*	N32°27′	E119°55′	3	Tai’an, Jiangsu
C4	*M. lutarioriparius*	N28°84′40″	E112°35′43″	48	Yuanjiang, Hunan
C5	*M. lutarioriparius*	N28°84′59″	E112°35′27″	24	Yuanjiang, Hunan
C6	*M. lutarioriparius*	N28°84′90″	E112°35′11″	35	Yuanjiang, Hunan
C7	*M. lutarioriparius*	N31°9′	E118°8′	17	Fanchang, Anhui
C8	*M. lutarioriparius*	N30°28′	E114°20′	78	Wuhan, Hubei
C9	*M. lutarioriparius*	N30°32′	E118°27′	113	Jingxian, Anhui

### DNA extraction

Fresh leaves from 50 *Miscanthus* individuals were collected and frozen in liquid nitrogen before being manually ground into a fine powder. Total genomic DNA was extracted following a modified cetyltrimethyl ammonium bromide (CTAB) method [46]. The leaves of *Miscanthus* are rich in phenols, therefore CTAB extraction solution was supplemented with 2% poly-N-vinylpyrrolidone (PVP) and 1% β-mercaptoethanol to purify the *Miscanthus* DNA. The concentration and quality of the extracted DNA was detected using 0.8% agarose gel electrophoresis and an ND-1000 spectrophotometer (Nano Drop), respectively.

### Enzyme digestion design

To identify the most appropriate enzymes for genomic digestion, we selected the *M. lutarioriparius* genome as the reference genome (https://www.nature.com/articles/s41467-020-18923-6). Based on the reference genome, suitable restriction enzyme combinations were predicted for the digestion by using DNassist [47]. To assess the efficiency of the predicted enzymes, the genome of Japanese rice (*Oryza sativa* L. subsp. *japonica*) was selected as a control.

### SLAF library construction and high-throughput sequencing

Using the identified restriction enzyme combination (*Eco*RV + *Sca*I), the total genomic DNA of each sample was digested. After adding an A-tail to the 3′ end of each digested fragment and ligating the fragment to the Dual-index sequencing adapter [48], each fragment was amplified through PCR. The amplicons were purified, mixed, and cut. The digested fragments were chosen as target segments. The libraries were selected and sequenced using an Illumina HiSeq TM 2500 platform (Biomarker Technologies Corporation), with a read length of 2 × 100 bp. The obtained sequenced reads were mapped based on the *M. lutarioriparius* reference genome for subsequent mutation analysis. To assess the accuracy of SLAF library construction, the Japanese rice genome was again used as a control.

### Development of SLAF tags and SNP markers

The Dual-index tags were used to classify the raw sequencing data by sample. Sequences reads from the same locus were grouped using similarity clustering [49]. In general, only high-depth fragments were selected in each cluster group, whereas low-depth segments were removed. Here, we first calculated the SLAF tags for each sample independently, and then all single-sample SLAF tags were clustered to derive population-wide SLAF tags. The positions of clean reads on the reference genome were compared, the sequencing depth of each sample was counted, and variations were detected. Each sequence was aligned with the reference sequence using bwa [47]. We used GATK [48] and SAMtools [49] to identify SNPs. The SNPs, identified by both methods were considered reliable. Of these reliable SNPs, those with integrity > 0.8 and MAF > 0.05 were considered highly consistent and were used for subsequent analyses.

### Genetic relationships among samples

We used admixture software [46] to determine the population structure of the 50 *Miscanthus* germplasms. We also performed principal components analysis (PCA) of the germplasms using cluster software [50]. PCAs was performed for linear transformations of variables to create orthogonal axes ordered by the proportion of variance explained [51]. A rooted phylogenetic tree based on our SNP data for these 50 germplasms was constructed using the neighbor-joining (NJ) method [52] in MEGA 5.0 software [53]. The phylogenetic tree based on our samples was constructed using the Kimura 2-parameter model with 1000 bootstrap replicates [54, 55].

## Data Availability

The *Miscanthus* SLAF tags and SNPs supporting the findings of this work are available within the paper and its Supplementary Information files. The raw date of *Miscanthus* sequence generated and analyzed during the current study are available from NCBI SRA data PRJNA786085, and the SRA records will be accessible with the following link: https://www.ncbi.nlm.nih.gov/sra/PRJNA786085.

## Abbreviations

AFLPs: Amplified fragment length polymorphisms
BAC: Bacterial artificial chromosome
CTAB: Cetyltrimethyl ammonium bromide
GBS: Genotyping by sequencing
ISSRs: Inter-simple sequence repeats
MAF: Minor allele frequency
NJ: Neighbor-joining
PCA: Principal components analysis
PVP: Poly-N- vinylpyrrolidone
RAD-seq: Restriction site-associated DNA sequencing
RAPD: Random amplified polymorphic DNA
RFLPs: Restriction fragment length polymorphisms
SLAF-seq: Specific-locus amplified fragment sequencing
SNPs: Single nucleotide polymorphisms
SSRs: Simple sequence repeats

## References

[1] Sang T (2011). Toward the domestication of lignocellulosic energy crops: learning from food crop domestication. J Integr Plant Biol.

[2] Sang T (2011). China's bioenergy potential. Glob Change Biol Bioenerg.

[3] Clifton-Brown J, Harfouche A, Casler MD, Dylan Jones H, Macalpine WJ, Murphy-Bokern D (2019). Breeding progress and preparedness for mass-scale deployment of perennial lignocellulosic biomass crops switchgrass, miscanthus, willow and poplar. Glob Change Biol Bioenerg.

[4] Liu W, Sang T. Potential productivity of the Miscanthus energy crop in the loess plateau of China under climate change. Environ Res Lett. 2013;8(4)4003. 10.1088/1748-9326/8/4/044003.

[5] Kirkpatrick J. Construction and analysis of the miscanthus genespace (doctoral dissertation, University of Illinois at Urbana-- Champaign). 2014. https://hdl.handle.net/2142/46925.

[6] Mitros T, Session AM, James BT, Wu GA, Belaffif MB, Clark LV (2020). Genome biology of the paleotetraploid perennial biomass crop Miscanthus. Nat Commun.

[7] De Vega J, Donnison I, Dyer S, Farrar K (2021). Draft genome assembly of the biofuel grass crop *Miscanthus sacchariflorus*. F1000Research.

[8] Miao J, Feng Q, Li Y, Zhao Q, Zhou C, Lu H (2021). Chromosome-scale assembly and analysis of biomass crop Miscanthus lutarioriparius genome. Nat Commun.

[9] Zhang G, Ge C, Xu P, Wang S, Cheng S, Han Y (2021). The reference genome of Miscanthus floridulus illuminates the evolution of Saccharinae. Nature Plants.

[10] Ge CX, Liu XM, Liu SM, Xu J, Li HF, Cui TT (2017). Miscanthus sp.: Genetic Diversity and Phylogeny in China. Plant Mol Biol Report.

[11] Li SS, Zhou HF, Chen WL, Yan J, Cai Z, Wei RX (2019). Population genetics and evolutionary history of Miscanthus species in China. J Syst Evol.

[12] Tang YM, Xiao L, Iqbal Y, Liao JF, Xiao LQ, Yi ZL (2019). Molecular cytogenetic characterization and phylogenetic analysis of four Miscanthus species (Poaceae). Comp Cytogenet.

[13] Olatoye MO, Clark LV, Labonte NR, Dong H, Dwiyanti MS, Anzoua KG (2020). Training Population Optimization for Genomic Selection in Miscanthus. G3 (Bethesda).

[14] Olatoye MO, Clark LV, Wang JP, Yang XP, Yamada T, Sacks EJ, et al. Evaluation of genomic selection and marker-assisted selection in Miscanthus and energycane. Mol Breed. 2019;39:171. 10.1007/s11032-019-1081-5.

[15] Graner A, Jahoor A, Schondelmaier J, Siedler H, Pillen K, Fischbeck G (1991). Construction of an RFLP map of barley. Theor Appl Genet.

[16] Paterson AH, Lander ES, Hewitt JD, Peterson S, Lincoln SE, Tanksley SD (1988). Resolution of quantitative traits into Mendelian factors by using a complete linkage map of restriction fragment length polymorphisms. Nature.

[17] Williams JG, Kubelik AR, Livak KJ, Rafalski JA, Tingey SV (1990). DNA polymorphisms amplified by arbitrary primers are useful as genetic markers. Nucleic Acids Res.

[18] John W, Michael M (1990). Fingerprinting genomes using PCR with arbitrary primers. Nucleic Acids Res.

[19] Vos P, Hogers R, Bleeker M, Reijans M, Van De Lee T, Hornes M (1995). AFLP: a new technique for DNA fingerprinting. Nucleic Acids Res.

[20] Litt M, Luty JA (1989). A hypervariable microsatellite revealed by in vitro amplification of a dinucleotide repeat within the cardiac muscle actin gene. Am J Hum Genet.

[21] Reddy MP, Sarla N, Siddiq EA (2002). Inter simple sequence repeat (ISSR) polymorphism and its application in plant breeding. Euphytica.

[22] Clark LV, Brummer JE, Glowacka K, Hall MC, Heo K, Peng JH (2014). A footprint of past climate change on the diversity and population structure of Miscanthus sinensis. Ann Bot.

[23] Ge C, Ai X, Jia S, Yang Y, Che L, Yi Z (2019). Interspecific genetic maps in Miscanthus floridulus and M. sacchariflorus accelerate detection of QTLs associated with plant height and inflorescence. Mol Gen Genomics.

[24] Spindel J, Wright M, Chen C, Cobb J, Gage J, Harrington S (2013). Bridging the genotyping gap: using genotyping by sequencing (GBS) to add high-density SNP markers and new value to traditional bi-parental mapping and breeding populations. Theor Appl Genet.

[25] Zeng B, Yan HD, Liu XC, Zang WJ, Zhang AL, Zhou SF, et al. Genome-wide association study of rust traits in orchardgrass using SLAF-seq technology. Hereditas. 2017;154:5. 10.1186/s41065-017-0027-3. 10.1186/s41065-017-0027-3 PMC5322626 28250720

[26] Liu LQ, Luo QL, Li HW, Li B, Li ZS, Zheng Q (2018). Physical mapping of the blue-grained gene from Thinopyrum ponticum chromosome 4Ag and development of blue-grain-related molecular markers and a FISH probe based on SLAF-seq technology. Theor Appl Genet.

[27] Jia Q, Tan C, Wang J, Zhang X-Q, Zhu J, Luo H (2016). Marker development using SLAF-seq and whole-genome shotgun strategy to fine-map the semi-dwarf gene ari-e in barley. BMC Genomics.

[28] Zhang Y, Zhang JP, Huang L, Gao AN, Zhang J, Yang XM (2015). A high-density genetic map for P genome of Agropyron Gaertn. based on specific-locus amplified fragment sequencing (SLAF-seq). Planta.

[29] Elshire RJ, Glaubitz JC, Sun Q, Poland JA, Kawamoto K, Buckler ES (2011). A robust, simple genotyping-by-sequencing (GBS) approach for high diversity species. PLoS One.

[30] Sun X, Liu D, Zhang X, Li W, Liu H, Hong W (2013). SLAF-seq: an efficient method of large-scale de novo SNP discovery and genotyping using high-throughput sequencing. Plos One.

[31] Zhang Y, Wang L, Xin H, Li D, Ma C, Ding X (2013). Construction of a high-density genetic map for sesame based on large scale marker development by specific length amplified fragment (SLAF) sequencing. BMC Plant Biol.

[32] Wei QZ, Wang YZ, Qin XD, Zhang YX, Zhang ZT, Wang J (2014). An SNP-based saturated genetic map and QTL analysis of fruit-related traits in cucumber using specific-length amplified fragment (SLAF) sequencing. BMC Genomics.

[33] Shan T, Pang S, Li J, Li X, Su L (2015). Construction of a high-density genetic map and mapping of a sex-linked locus for the brown alga Undaria pinnatifida (Phaeophyceae) based on large scale marker development by specific length amplified fragment (SLAF) sequencing. BMC Genomics.

[34] Jiang B, Liu WR, Xie DS, Peng QW, He XM, Lin YE, et al. High-density genetic map construction and gene mapping of pericarp color in wax gourd using specific-locus amplified fragment (SLAF) sequencing. BMC Genomics. 2015;16:1035. 10.1186/s12864-015-2220-y. 10.1186/s12864-015-2220-y PMC4673774 26647294

[35] Shang JL, Li N, Li NN, Xu YY, Ma SW, Wang JM (2016). Construction of a high-density genetic map for watermelon (*Citrullus lanatus* L.) based on large-scale SNP discovery by specific length amplified fragment sequencing (SLAF-seq). Scientia Horticulturae.

[36] Gong DP, Huang L, Xu XH, Wang CY, Ren M, Wang CK, et al. Construction of a high-density SNP genetic map in fluecured tobacco based on SLAF-seq. Mol Breed. 2016;36(100) 10.1007/s11032-016-0514-7.

[37] Dong ZM, Chen L, Li Z, Liu NX, Zhang SC, Liu J, et al. Identification and molecular mapping of the semi-dwarf locus (sdf-1) in soybean by SLAF-seq method. Euphytica. 2020;216:103. 10.1007/s10681-020-02633-7.

[38] Liu T, Guo L, Pan Y, Zhao Q, Wang J, Song Z (2016). Construction of the first high-density genetic linkage map of Salvia miltiorrhiza using specific length amplified fragment (SLAF) sequencing. Sci Rep.

[39] Chen S, Huang Z, Dai Y, Qin S, Gao Y, Zhang L (2013). The development of 7E chromosome-specific molecular markers for Thinopyrum elongatum based on SLAF-seq technology. Plos One.

[40] Xia C, Chen LL, Rong TZ, Li R, Xiang Y, Wang P (2015). Identification of a new maize inflorescence meristem mutant and association analysis using SLAF-seq method. Euphytica.

[41] Zhao X, Huang L, Zhang X, Wang J, Yan D, Li JM (2016). Construction of high-density genetic linkage map and identification of flowering-time QTLs in orchardgrass using SSRs and SLAF-seq. Sci Rep.

[42] Chen S & Renvoize SA. Miscanthus Andersson, Öfvers. Kongl. Vetensk.-Akad. Förh. 12: 165, 1855, (2006). Flora of China 22, 581–583.49.

[43] Zheng C, Xue S, Xiao L, Iqbal Y, Sun GR, Duan MJ, et al. “Two-steps” seed-derived plugs as an effective propagation method for the establishment of Miscanthus in saline–alkaline soil. GCB Bioenergy. 2021;3. 10.1111/gcbb.12820.

[44] Yan J, Zhu CY, Liu W, Luo F, Mi J, Ren YJ (2015). High photosynthetic rate and water use efficiency of *Miscanthus lutarioriparius* characterize an energy crop in the semiarid temperate region. GCB Bioenergy.

[45] Xue S, Guo MQ, Iqbal Y, Liao JF, Yang S, Xiao L (2020). Mapping current distribution and genetic diversity of the native Miscanthus lutarioriparius across China. Renew Sustain Energy Rev.

[46] Xia YN, Xu J, Duan JY, Liu QB, Huang HM, Yi ZL (2019). Transgenic Miscanthus lutarioriparius that co-expresses the cry 2Aa# and Bar genes. Can J Plant Sci.

[47] Parkinson J, Blaxter M (2009). Expressed sequence tags: an overview. Methods Mol Biol.

[48] Kanazin V, Talbert H, See D, Decamp P, Nevo E, Blake T (2002). Discovery and assay of single nucleotide polymorphisms in barley (Hordeum vulgare). Plant Mol Biol.

[49] Jones JC, Fan SH, Franchini P, Schartl M, Meyer A (2013). The evolutionary history of Xiphophorus fish and their sexually selected sword: a genome-wide approach using restriction site-associated DNA sequencing. Mol Ecol.

[50] Sheng J, Zheng X, Wang J, Zeng X, Zhou F, Jin S (2017). Transcriptomics and proteomics reveal genetic and biological basis of superior biomass crop Miscanthus. Sci Rep.

[51] Kim C, Tang H, Paterson AH (2009). Duplication and divergence of grass genomes: integrating the Chloridoids. Trop Plant Biol.

[52] Kim C, Zhang D, Auckland SA, Rainville LK, Jakob K, Kronmiller B (2012). SSR-based genetic maps of Miscanthus sinensis and M. sacchariflorus, and their comparison to sorghum. Theor Appl Genet.

[53] Phillips SM, Dubery IA, Van Heerden H (2013). Molecular characterisation of two homoeologous elicitor-responsive lipin genes in cotton. Mol Gen Genomics.

[54] Mahadani AK, Awasthi S, Sanyal G, Bhattacharjee P, Pippal S. Indel-K2P: a modified Kimura 2 parameters (K2P) model to incorporate insertion and deletion (Indel) information in phylogenetic analysis. Cyber Phys Syst. 2021. 10.1080/23335777.2021.1879274.

[55] Kaur J, Bhambri P, Gupta OP (2012). Distance based phylogenetic trees with bootstrapping. Int J Comp Appl.

